# Preparation of High Mechanical Strength Chitosan Nanofiber/NanoSiO_2_/PVA Composite Scaffolds for Bone Tissue Engineering Using Sol–Gel Method

**DOI:** 10.3390/polym14102083

**Published:** 2022-05-20

**Authors:** Wei Ma, Sihan Zhang, Chong Xie, Xing Wan, Xiaofeng Li, Kebing Chen, Guanglei Zhao

**Affiliations:** 1 State Key Laboratory of Pulp and Paper Engineering, School of Light Industry and Engineering, South China University of Technology, Guangzhou 510641, China; 201921027612@scut.edu.cn (W.M.); 201710104778@mail.scut.edu.cn (S.Z.); 201810107072@mail.scut.edu.cn (C.X.); wanxing0831@163.com (X.W.); 2 Department of Spine Surgery, The Sixth Affiliated Hospital of Sun Yat-sen University, 26 Erheng Road, Yuan Village, Guangzhou, 510655, China

**Keywords:** bone tissue engineering, nanofiber, chitosan, sol–gel, PVA

## Abstract

The majority of chitosan-based bone tissue engineering (BTE) scaffolds have the problem of poor mechanical properties. However, modifying chitosan with conventional silane coupling agents to improve the mechanical properties of scaffolds will introduce additional complications, including cytotoxicity and poor biocompatibility. In this study, two types of organic–inorganic composite scaffolds (F-A-T0/T3/T5 and F-B-T5-P0/P0.5/P1.5/P2.5) were prepared using chitosan nanofibers (CSNF) prepared by the beating-homogenization method, combined with the sol–gel method, and further introduced polyvinyl alcohol (PVA). The F-A-T3 and F-B-T5-P1.5 exhibited interconnected pore and surface nanofibers structures, high porosity (>70%), outstanding swelling properties, and a controllable degradation rate. The Young’s modulus of TEOS: 5.0% (*w*/*w*), PVA: 1.5% (*w*/*w*) chitosan fiber scaffold is 8.53 ± 0.43 MPa in dry conditions, and 237.78 ± 8.86 kPa in wet conditions, which is four times that of F-A-T5 and twice that of F-B-T5-P0. Additionally, cell (MC3T3-E1) experiments confirmed that the two composite scaffolds had great cytocompatibility and were predicted to be used in the future in the field of BTE scaffolds.

## 1. Introduction

Bone is a high-density connective tissue with an intrinsic ability to repair itself; however, it is unable to repair defects that exceed the critical size (>2 cm) [1,2]. Traditional bone strategies, such as autogenous bone transplantation, allogeneic bone transplantation, or physiologically inert metal devices, carry the risk of infection and complications [3,4]. In recent years, the advent of bone tissue engineering (BTE) has significantly promoted the development of materials for bone defect repair to avoid these risks effectively [5]. Among the three components of BTE (seed cells, growth factors, and scaffold materials), biocompatibility, porosity, the roughness of the surface, good mechanical properties, and biodegradability of the scaffold materials are critical for BTE [6,7,8,9,10], as they provide space for cell proliferation, development, and differentiation [11]. Due to the complexity of bone composition and structure, it makes sense to combine several materials to create composite scaffolds during the scaffold manufacturing process to enhance the bioactivity and structural bionics of the scaffolds. Organic–inorganic scaffolds have been proven to combine the advantages of polymers and inorganics, enhancing mechanical strength, increasing bioactivity, regulating pore structure, and encouraging vascularization [12,13,14].

The sol–gel method has simple synthesis conditions, can form a stable network with controllable reaction activity in an organic matrix, realize the control of the morphology and the size of the nano-scale, and conveniently and effectively prepare the organic–inorganic composite scaffold material [15,16]. Briefly, the sol–gel process consists of two stages: solution and gelation, with the sol representing a colloidal suspension of solid particles and the gel representing a linked network of solid particles [13,15]. Tetraethyl orthosilicate (TEOS) is frequently used as a precursor for the sol–gel method in bone tissue engineering. It forms a highly controlled network with simple control of the synthesis conditions and is easily mixed with biomolecules or cells [15]. The nano-silica particles produced during the hydrolysis and polycondensation of TEOS can be used to increase the surface roughness of the composite material, induce osteoblast differentiation, inhibit osteoclast differentiation, promote osteoblast adhesion, stimulate osteogenic gene expression, and significantly improve the composite material’s mechanical properties [17].

Chitosan (CS) is the most abundant polymer in nature, second only to cellulose [18], and is similar to glycosaminoglycans present in the extracellular matrix (ECM). CS is an ideal material for the organic component due to its outstanding biocompatibility, biodegradability, and bioactivity [18,19]. Polyvinyl alcohol (PVA) is a type of polymer that exhibits hydrophilicity, biocompatibility, and biodegradability [20]. In the presence of concentrated hydrochloric acid, network silica and PVA are chemically crosslinked using the sol–gel method, which significantly improves the mechanical properties of hybrid materials [21,22].

Chitosan nanofibers (CSNF) have recently received a lot of attention due to their mechanical strength properties, morphological similarity to ECM, and nano-size effect [23,24,25,26]. Technologies such as electrospinning and thermally induced phase separation (TIPS) have been widely used to develop nanoscale chitosan materials [27,28]. However, electrospinning and TIPS have limitations, including using toxic solvents, process complexity, size non-uniformity, and low nanofiber yields [28,29,30,31]. Our group developed a novel chitosan nanofiber with a high surface charge and a long fiber length using the beating homogenization method, which demonstrates an effective method for the efficient and industrial manufacturing of chitosan nanofibers without the use of organic solvents. The preparation of chitosan nanofiber-based organic–inorganic scaffolds by the sol–gel method has considerable potential in the field of bone tissue engineering scaffolds.

In this study, two types of organic–inorganic composite scaffolds (F-A-T0/T3/T5 and F-B-T5-P0/P0.5/P1.5/P2.5) were prepared using CSNF prepared by the beating homogenization method, combined with the sol–gel method, and further introduced PVA. The scaffolds were characterized by field emission scanning electron microscopy (FESEM), atomic force microscopy (AFM), Fourier transform infrared spectrometer (FT-IR), X-ray diffractometer (XRD), porosity, swelling properties, mechanical properties, in vitro degradation and cell experiments. On the structure and characteristics of the scaffolds, the impacts of chitosan type, acidic and alkaline preparation conditions, and PVA concentration were examined.

## 2. Materials and Methods

### 2.1. Materials

Chitosan regenerated fiber (CSF, ≥95% deacetylated, ≥1000 mPa·s viscosity) prepared by wet spinning, was purchased from Shandong Laizhou Highly Bio-chemicals. Chitosan (Laizhou, China), (≥95% deacetylated, 100–200 mPa·s viscosity) and Polyvinyl alcohol (MW-27000 g/mol) were obtained from Shanghai Macklin Biochemical Co., Ltd. (Shanghai, China). The culture media included fetal bovine serum (FBS, Gibco Life Technology, Waltham, MA, USA), MEM basic (MEM, minimum essential medium, Gibco), penicillin-streptomycin (PS, 10,000 U/mL, and 10,000 μg/mL, respectively, Gibco), 0.25% trypsin-EDTA (Gibco), phosphate-buffered saline (PBS, pH 7.4, Gibco), Cell Counting Kit-8 (CCK-8, Biosharp Life Sciences, Hefei, China), and Calcein-AM/PI Double Staining Kit (Calcein AM/PI, Dalian Meilun Biotechnology, Dalian, China). The water used in the preparation process was obtained using a pure water machine (Purelab, ELGA, Partille, Sweden). Other reagents used, such as TEOS, acetic acid, ethanol, hydrochloric acid, and ammonia solution, were commercially available and of analytical grade.

### 2.2. Preparation of CSNF

**Figure 1:**
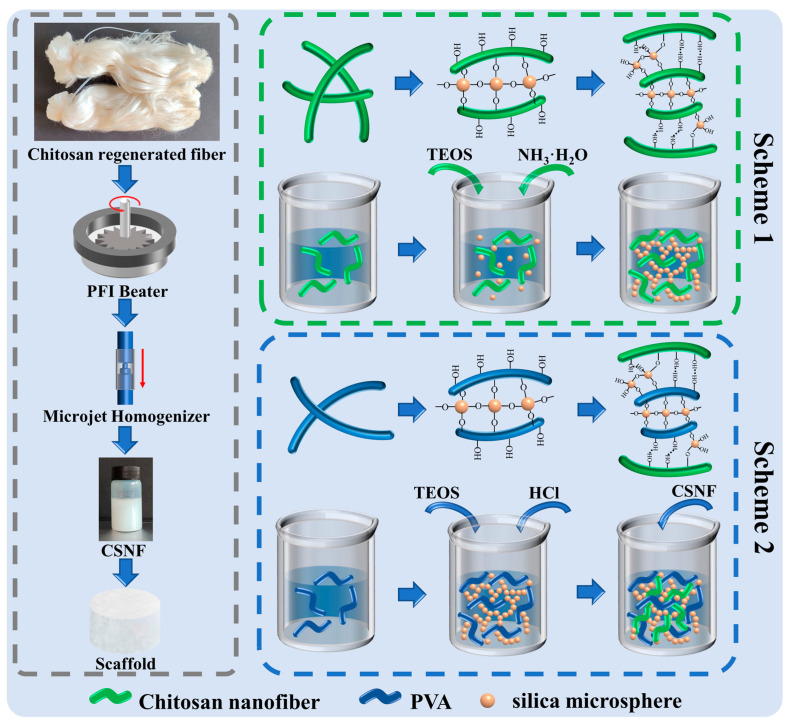
Schematic drawing of chitosan nanofiber/nanoSiO_2_ (**Scheme 1**), and chitosan nanofiber/nanoSiO_2_/PVA (**Scheme 2**) scaffolds preparation steps.

According to Zhang’s research [31], the chitosan regenerated fibers were first cut into 2–5 mm lengths using a cutting mill (CM200, GRINDER, Beijing, China) to maintain a uniform morphology. Then the pulp was milled at 30,000 rpm with a PFI refiner set to 3.33 N/mm pressure to further decrease the fiber length. Finally, uniform diameter CSNF can be obtained by homogenizing the slurry ten times at 100 bar with a microflow nanohomogenizer (Nano DeBEE Next Generation Homogenizers, Nano BEE, South Easton, MA, USA), as shown in Figure 1.

### 2.3. Preparation of P-A-T3 and F-A-T0/T3/T5 Scaffolds

The preparation route of the chitosan nanofiber/nanoSiO_2_ scaffolds is shown in Scheme 1 of Figure 1, the composition is listed in Table 1, and the exact preparation steps are as follows:

(1) Chitosan powder solution was made by weighing a certain amount of chitosan powder, adding deionized water and a certain amount of glacial acetic acid, and stirring with a magnetic stirrer for 3 h;
(2) The homogenized CSNF was adjusted to a dispersion concentration of 2.5% (*w*/*w*) by centrifugation and the addition of deionized water;
(3) A certain amount of TEOS was dissolved 1 mL of ethanol and then added to the dispersion of (1) and (2), which were then stirred for 1 h using a magnetic stirrer to ensure equitable mixing. Then, a certain amount of ammonia water (V_NH3·H2O_:V_CSNF/CS dispersion_ = 1:10) was added and stirred for 24 h at 30 °C;
(4) Pour the fully reacted dispersion into a 48-well plate, allowing it to stand for 3 h, and then freeze-dry the scaffold. Finally, soak then wash with deionized water three times, and freeze-dry again to achieve the final composite scaffolds.

### 2.4. Preparation of F-B-T5-P0/P0.5/P1.5/P2.5 Scaffolds

The preparation route of the chitosan nanofiber/nanoSiO_2_/PVA scaffolds is shown in Scheme 2 of Figure 1, the composition is listed in Table 1, and the exact preparation steps are as follows:

(1) Weigh a certain amount of PVA and add it into the deionized water, and continue heating and stirring at 80 °C for 5 h until the PVA is completely dissolved;
(2) A certain amount of TEOS was dissolved in 1 mL of ethanol and then added to the PVA solution, which was then stirred for 1 h using a magnetic stirrer to ensure equitable mixing. Then, a certain amount of 10% (*w*/*w*) HCl (1 drop/10 mL PVA solution) was added and stirred for 12 h at 60 °C;
(3) To the thoroughly reacted dispersion, an equal quality of 5.0% (*w*/*w*) CSNF dispersion was added and stirred for 12 h at 60 °C to ensure equitable mixing;
(4) Pour the fully reacted dispersion into a 48-well plate, allowing it to stand for 3 h, and then freeze-dry the scaffold. Finally, soak then wash with deionized water three times, and freeze-dry again to achieve the final composite scaffolds.

### 2.5. Characterization of the Scaffolds

The porous structure of the scaffolds was analyzed using field emission scanning electron microscopy (FESEM, Merlin Compact, Carl ZEISS, Jena, Germany). After cutting the scaffolds, an EMS300T sputter coating machine (EMSDIASUM, Hatfield, UK) was used to plate 5–20 nm platinum/palladium (Pt/Pd) on the surface of the scaffolds, and then FESEM was used to image under 5 kV voltage. A Nano Measure was used to measure the pore size distribution with at least 100 holes.

A relatively flat piece was cut from the scaffolds, and the double-sided tape was used to adhere it to the mica sheet’s surface. Atomic force microscopy (AFM, Multimode 8, Bruker, Billerica, MA, USA) was used to examine the surface morphology of the composite scaffolds. Topographic and phase images were recorded simultaneously using a standard silicon tip on a cantilever beam. 

A Fourier transform infrared (FT-IR) spectrophotometer (TENSOR27/HYPERION, Bruker, Bremen, Germany) was used to perform FT-IR spectroscopy on the scaffolds in the range of 400–4000 cm^−1^.

A multi-position automatic sampling X-ray diffractometer (X’pert Powder, PANalytical, Almelo, The Netherlands) was used for the phase analysis of the scaffolds. The scanning range (2θ) was 5–60°, the scanning step was 0.013°, and the scanning speed was 12°/min.

### 2.6. Porosity, Swelling, and Degradation Studies of Scaffolds

For the porosity test, the volume (V) and weight (W_1_) of the dry scaffolds were measured, and then after completely immersing the scaffolds in ethanol for 24 h, the final weight of the scaffolds (W_2_) was measured. This equation was used to calculate the porosity of the scaffolds.

Porosity (%) = (W_2_ − W_1_)/(ρ_ethanol_·V), (1)

For the swelling test, the scaffolds were immersed in PBS for different time intervals, and excess liquid was absorbed from the surface with filter paper. The quality of the scaffolds in the dry (W_ds_) and swollen (W_ss_) conditions were measured with an analytical balance. The swelling rate (%) of the scaffolds was calculated by the following formula.

Swelling ratio (%) = [(W_ss_ − W_ds_)/W_ds_] × 100, (2)

For the degradation test, the scaffolds were accurately weighed (W_0_) and immersed in PBS containing lysozyme (enzyme activity 10,000 U/L), and PBS was refreshed every two days for a total of twenty-eight days. The scaffolds were removed at 7, 14, 21, and 28 days, washed with pure water three times, lyophilized, and weighed (W_1_). The degradation rate can be calculated by the following formula.

W_loss_ (%) = [(W_0_ − W_1_)/W_0_] × 100, (3)

### 2.7. Compressive Strength of Scaffolds

The mechanical properties of the scaffolds with a diameter of 10 mm and a height of 10 mm were measured using a universal tensile tester (INSTRON 3300, Instron, Norwood, MA, USA) under dry and wet conditions. Before testing, the wet samples were immersed in PBS for several hours. When the test failed or the specimen’s height was decreased to 70% of its original height, the stress–strain curves of all scaffolds were immediately recorded. Young’s modulus is the slope of the straight line preceding the stress–strain curve.

### 2.8. MC3T3-E1 Cells Compatibility and Proliferation Assay

Mouse calvaria-derived pre-osteoblastic cells (MC3T3-E1) were used to evaluate the biocompatibility of the composite scaffolds [31]. The cells were cultured in MEM supplemented with 10% FBS and 1% PS and incubated at 37 °C in a 5% CO_2_ atmosphere. The medium was changed every other day to maintain the cell conditions. The scaffolds were cut into cylindrical samples with a diameter of ~10 mm and a thickness of ~2 mm. The as-prepared samples were sterilized with UV light for 3 h, immersed in 75% ethanol for 3 h, washed three times with PBS, transferred to a 24-well plate, immersed in a culture medium for 3 h, and then semi-dried. The cells were seeded at a density of 1 × 10^4^ cells/well. The samples were incubated at 37 °C and 5% CO_2_ for 2 h to ensure that the majority of cells adhered to the scaffolds, and then 500 μL of culture medium was added to each well. The culture medium was replaced every other day.

On days 1, 3, 5, and 7, the viability of the cells was determined using CCK-8. The optical density (OD) value was measured at 450 nm using a multifunctional microplate reader. Each group underwent three independent tests. Additionally, on days 1, 3, 5, and 7, live/dead cells were stained with Calcein AM/PI. After incubation for 30 min at 37 °C and under 5% CO_2_ cell morphology was observed using an inverted fluorescence microscope.

## 3. Results

### 3.1. Structural Analyses of the Scaffolds

**Figure 2:**
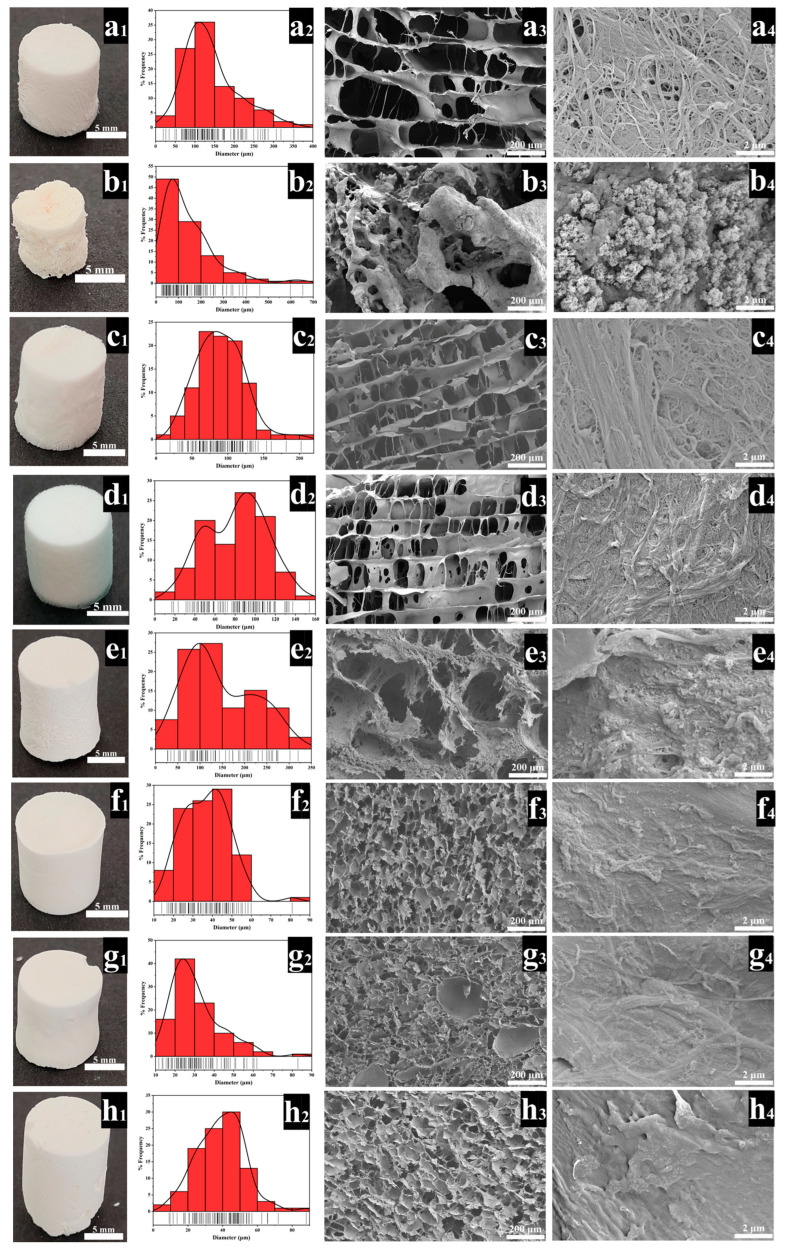
The actual image, the pore size distribution, the SEM image magnified 100 times, and the scaffolds magnified 10 K times, respectively, are shown from left to right: (**a1**–**a4**) F-A-T0, (**b1**–**b4**) P-A-T3, (**c1**–**c4**) F-A-T3, (**d1**–**d4**) F-A-T5, (**e1**–**e4**) F-B-T5-P0, (**f1**–**f4**) F-B-T5-P0.5, (**g1**–**g4**) F-B-T5-P1.5, (**h1**–**h4**) F-B-T5-P2.5.

Highly porous and surface nanofibrous microstructures are desired characteristics of BTE scaffolds, which are conducive to cell adhesion and growth, as well as for the diffusion of ions and nutrients [32,33]. As shown in Figure 2, all scaffolds exhibited a highly interconnected pore structure. The pore size distribution of F-A-T0 (Figure 2a1–a4) is between 50 and 250 μm, the nanofibers on the scaffold’s surface are interwoven, and the diameter of a single nanofiber is between 30 and 300 nm. The pore size distribution of P-A-T3 (Figure 2b1–b4) is between 30 and 300 μm, and the pore distribution is uneven. On the surface of P-A-T3 (Figure 2b4), there are no nanofibers, simply a high number of NSiO_2_ particles with a diameter of roughly 10 nm. The pore size distribution of F-A-T3 (Figure 2c1–c4) is more uniform, ranging between 50–150 μm, the scaffold surface is interwoven with nanofibers, and a large number of NSiO_2_ particles are deposited, significantly increasing the surface roughness. The uniform pore size is more favorable for cell adhesion and growth [34], which may be due to the formation of an inorganic network, which increases the steric barrier between CSNF, resulting in improved dispersion and, ultimately, a uniform pore microstructure [35]. The pores on the surface of F-A-T5 (Figure 2d1–d4) were significantly reduced, showing an unconnected structure. This could be because, as the TEOS concentration increased, more NSiO_2_ was connected via covalent bonds, which caused greater resistance to the growth of ice crystals during freeze-drying, and eventually resulted in the reduction of pores and unconnected structure. The pore size distribution of F-B-T5-P0 (Figure 2e2) was predominantly between 100 and 250 μm, but the pore size distribution of F-B-T5-P0.5 (Figure 2f2) was between 10 and 60 μm, demonstrating that the addition of PVA could drastically lower the pore size of the scaffold. This could be owing to PVA’s high viscosity, which prevents water molecule diffusion during freezing and results in the development of small ice crystals, gradually reducing the scaffold pore size [35]. The pore size distributions of F-B-T5-P1.5 (Figure 2g2) and F-B-T5-P2.5 (Figure 2h2) are in the range of 10–60 μm, indicating that the PVA-modified scaffolds have higher mechanical properties. The smaller pore size enables nutrient adsorption on the surface, which is critical during the cell’s differentiation phase [22]. As demonstrated in Figure 2d4,e4, the nanofibers on the surface of F-B-T5-P0 are not very visible, which may be due to the partial dissolution of CSNF in hydrochloric acid. As illustrated in Figure 2f4,g4,h4, the nanofiber structure and NSiO_2_ are also not very visible, which may be due to PVA covering the surface of nanofibers and NSiO_2_.

**Figure 3:**
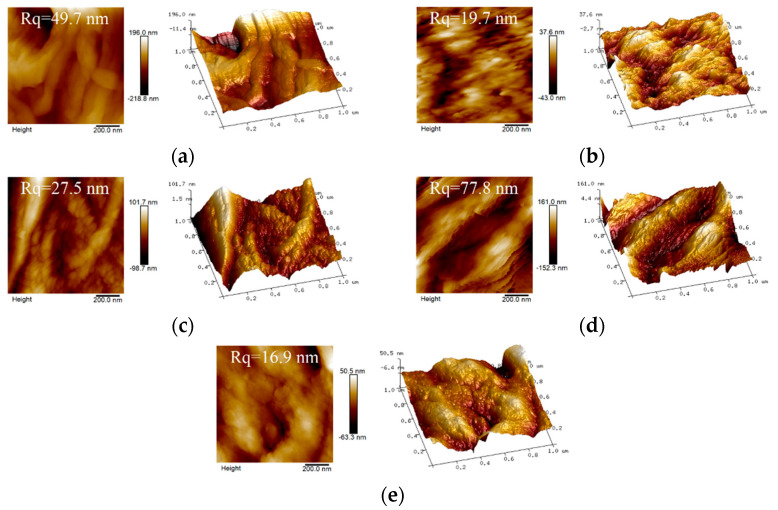
AFM images of (**a**) F-A-T0, (**b**) P-A-T3, (**c**) F-A-T3, (**d**) F-B-T5-P0, (**e**) F-B-T5-P1.5 scaffolds.

The rough surface structure of the scaffold material is beneficial to effective cell adhesion and growth [36]. As shown in Figure 3a, the F-A-T0 scaffold surface roughness value is around 49.7 nm, and the nanofiber structure with a diameter of about 50–200 nm can be seen in the 3D image. Because there was no nanofiber structure but NSiO_2_ particles on the surface, the surface roughness of P-A-T3 (Figure 3b) was the lowest, at about 19.7 nm. F-A-T3 (Figure 3c) has a surface roughness of 27.5 nm, which is halfway between F-A-T0 and P-A-T3. Because F-A-T3 has a nanofiber structure, the NSiO_2_ particles loaded on the surface will reduce the surface roughness and increase the specific surface area. F-B-T5-P0 (Figure 3d) exhibits the highest surface roughness value of about 77.8 nm. This could be due to the partial dissolution of CSNF by hydrochloric acid, which makes the overlap between CSNF more compact (similar to welding) and will not be destroyed during freeze-drying, resulting in a higher topography at the overlap. In addition, F-B-T5-P1.5 (Figure 3e) has a surface roughness value of around 16.9 nm. This is because CSNF and NSiO_2_ are coated with PVA, hence reducing the surface roughness.

### 3.2. Characterization Analyses of the Scaffolds

**Figure 4:**
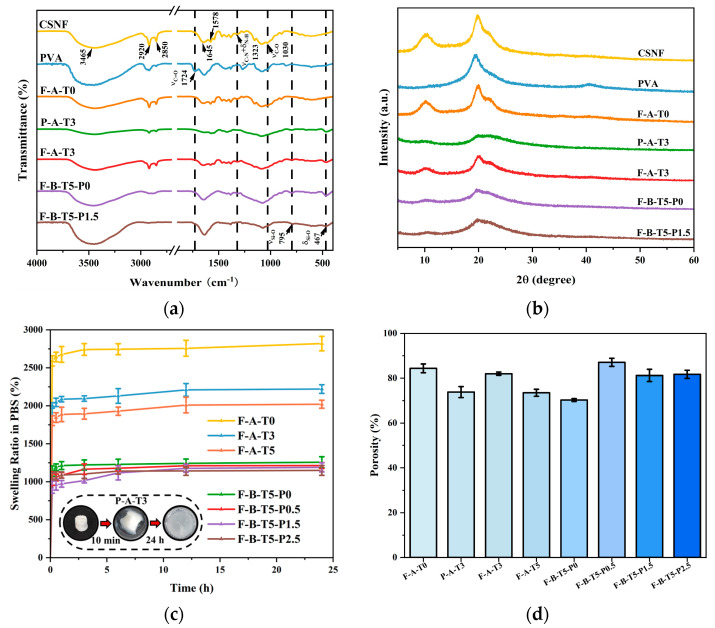
(**a**) FT-IR and (**b**) XRD of CSNF, PVA, F-A-T0/T3, P-A-T3, and F-B-T5-P0/P1.5. (**c**) swelling ration and (**d**) porosity of scaffolds.

The FT-IR spectra are shown in Figure 4a. The infrared spectrum of the CSNF, F-A-T0, P-A-T3, F-A-T3, F-B-T5-P0/P1.5 all showed typical absorption peaks of chitosan at 3465 cm^−1^ (ν_N-H_ + ν_O-H_), 2920 cm^−1^ and 2850 cm^−1^ (ν_C-H_), 1645 cm^−1^ (amide I band, ν_C=O_), 1578 cm^−1^ (amide II band, δ_N-H_) and 1323 cm^−1^ (amide III bands, ν_C-N_ + δ_N-H_) [37]. P-A-T3, F-A-T3, and F-B-T5-P0/P1.5 exhibited typical absorption peaks of silicon dioxide at 800 cm^−1^ and 467 cm^−1^ (ν_Si-O_ + δ_Si-O_). The Si-O-C bonds were in the range 1120–1080 cm^−1^, which overlaps with the Si-O-Si bond absorption interval [21,38]. This confirms that NSiO_2_ is successfully covalently linked to CSNF within F-A-T0/T3 scaffolds. Moreover, the characteristic absorption peak of PVA at 1724 cm^−1^ was observed to be disappeared in F-B-T5-P1.5 [39], indicating that the polycondensation interaction between PVA and NSiO_2_ occurred.

Figure 4b shows the XRD diffraction patterns of CSNF, PVA, F-A-T0, P-A-T3 F-A-T3, F-B-T5-P0, and F-B-T5-P1.5 scaffolds. In the XRD spectra of the P-A-T3, F-A-T3, and F-B-T5-P1.5 scaffolds, it could be seen that the peak of chitosan at 20° overlapped with the peak of silica at 22° and the peak of PVA at 19.5° to form a broad peak [40,41]. Additionally, the peak intensities of chitosan in these composite scaffolds were all reduced when compared to F-A-T0, which is consistent with Dodda’s results that NSiO_2_ and PVA were spread throughout the CSNF, eliminating the fine nanostructures that existed between the initial CSNF [40].

Good swelling performance is important for BTE scaffolds, which is conducive to the diffusion of nutrients and the adhesion and growth of cells [42]. As shown in Figure 4c, the P-A-T3 expanded greatly after 10 min in PBS and lost its morphology after 24 h, indicating that it could not meet the application requirements for BTE scaffolds. Other scaffolds swelled fast within 10 min of the experiment’s start, and even after 24 h of immersion in PBS, they retained sufficient strength and stability, demonstrating the advantages of CSNF over conventional chitosan materials. Furthermore, as the concentration of PVA was increased, the swelling property of the scaffolds gradually decreased, indicating that the swelling property of the scaffolds can be regulated by altering the concentration of PVA to meet the requirements of practical applications. This may be because the surface roughness of the scaffold reduces as the PVA concentration increases.

The highly interconnected porous structure is the ideal feature of BTE scaffolds, which is conducive to nutrition and ion diffusion [32,36]. Research has shown that scaffold materials with a total porosity greater than 60% are suitable for bone tissue regeneration applications [43]. As shown in Figure 4d, all scaffolds had a porosity of more than 70%. The porosity of the scaffolds increased significantly with the introduction of PVA but decreased with the increase in the concentration of PVA. This may be due to the high viscosity of PVA and the chemical crosslinking between PVA and NSiO_2_, which results in the formation of a more dense network structure between CSNF, NSiO_2_, and PVA, which inhibits the growth of ice crystal size during freezing, thereby decreasing porosity, and some studies have demonstrated that the addition of PVA increases porosity [44,45].

### 3.3. Compressive Strength of Scaffolds

**Figure 5:**
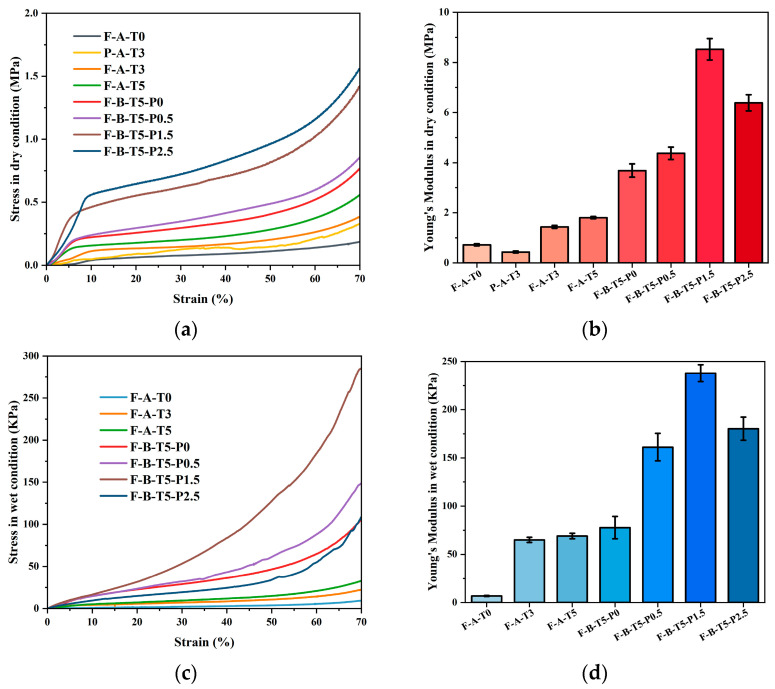
Compressive strength. (**a**) The compressive stress–strain curve in dry conditions. (**b**) Young’s modulus in dry conditions. (**c**) The compressive stress–strain curve in wet conditions. (**d**) Young’s modulus in wet conditions.

Mechanical properties are crucial to scaffolds for BTE, as the scaffolds operate as a temporary support matrix at the site of the bone deficit until new tissue grows [13]. We tested the compressive strength of the scaffolds under dry and wet conditions, as shown in Figure 5. F-A-T3 has a compressive strength of 382.18 kPa and an elastic modulus of 1.43 ± 0.06 MPa in the dry conditions, and compressive strength of 22.33 kPa, and Young’s modulus of 65.32 ± 2.65 kPa in the wet state, which is more than that of F-A-T0 and P-A-T3, and comparable to that of cancellous bone [46]. Due to the absence of CSNF interwoven network in P-A-T3, the scaffold retains its strength in dry conditions but entirely loses its morphology after a few minutes in PBS, making it impossible to assess its mechanical properties in the wet conditions. This suggests that CSNF is the basic framework of scaffolds, NSiO_2_ particles provide rigidity, and the organic–inorganic network is intertwined, endowing scaffolds with higher comprehensive mechanical properties. Compared with F-A-T5, F-B-T5-P0 exhibited superior mechanical properties, which may be a result of the partial dissolving of CSNF by concentrated hydrochloric acid, resulting in a stronger binding between CSNF. The compressive strength of the F-B-T5-P0/0.5/1.5/2.5 scaffolds increased initially and then dropped as the PVA concentration increased. This could be because PVA is a long-chain polymer that confers greater toughness on the scaffold, and when used in excess, the scaffold’s flexibility increases, but its mechanical rigidity reduces. Compressive strength and Young’s modulus of F-B-T5-P1.5 were 1.42 MPa and 8.53 ± 0.43 MPa in dry conditions, and 284.82 kPa and 237.78 ± 8.86 kPa in wet conditions, which met the mechanical requirements for cancellous bone graft [32].

### 3.4. Degradation Rate and Cytocompatibility of Scaffolds

P-A-T3 lost its scaffold shape quickly when immersed in water. Therefore, there is no need for degradation rate and cytocompatibility testing of it.

**Figure 6:**
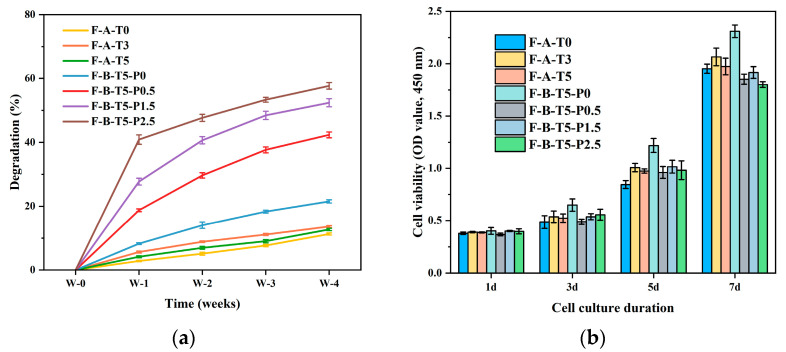
(**a**) Degradation and (**b**) CCK-8 assay of F-A-T0/T3/T5 and F-B-P0/P0.5/P1.5/P2.5.

The degradation rate of scaffolds is crucial for cell adhesion and proliferation. A faster degradation rate will weaken the support of the structure, and a slower degradation rate will limit the space for cell growth [47,48]. As shown in Figure 6a, after seven days, the scaffolds degradation rates of F-A-T0/T3/T5 and F-B-T5-P0/P0.5/P1.5/P2.5 were 2.82 ± 0.21%, 5.66 ± 0.32%, 4.16 ± 0.32%, 8.28 ± 0.27%, 18.76 ± 0.43%, 27.74 ± 1.07%, and 40.93 ± 1.47%, respectively. Additionally, the degradation rate dropped as the degradation time increased. The degradation rate of scaffolds was positively associated with the concentration of PVA, which may be explained by not only lysozyme degradation, but also by PVA dissolving in water. This demonstrates that the degradation rate of the composite scaffolds may be altered by adjusting the concentration of PVA added according to the actual requirements.

Cytocompatibility is an important consideration factor for the practical application of scaffold materials [13]. MC3T3-E1 cells were inoculated on F-A-T0/T3/T5 and F-B-T5-P0/P0.5/P1.5/P2.5 and cultured for 1, 3, 5, and 7 days. The CCK-8 was used to evaluate the cell viability of the scaffolds, as shown in Figure 6b. F-A-T3 had a higher OD value than F-A-T0/T5, indicating that the sol–gel formed NSiO_2_ network and the more interconnected pore structure provides more favorable circumstances for cell adsorption and growth. The OD value of F-B-T5-P0 is higher than that of F-B-T5-P0.5/P1.5/P2.5, which may be attributed to the fact that the addition of PVA reduces the scaffold’s pore size, which is detrimental to cell growth within the scaffolds. However, the OD value of all scaffolds increased, indicating that their cytotoxicity is low.

**Figure 7:**
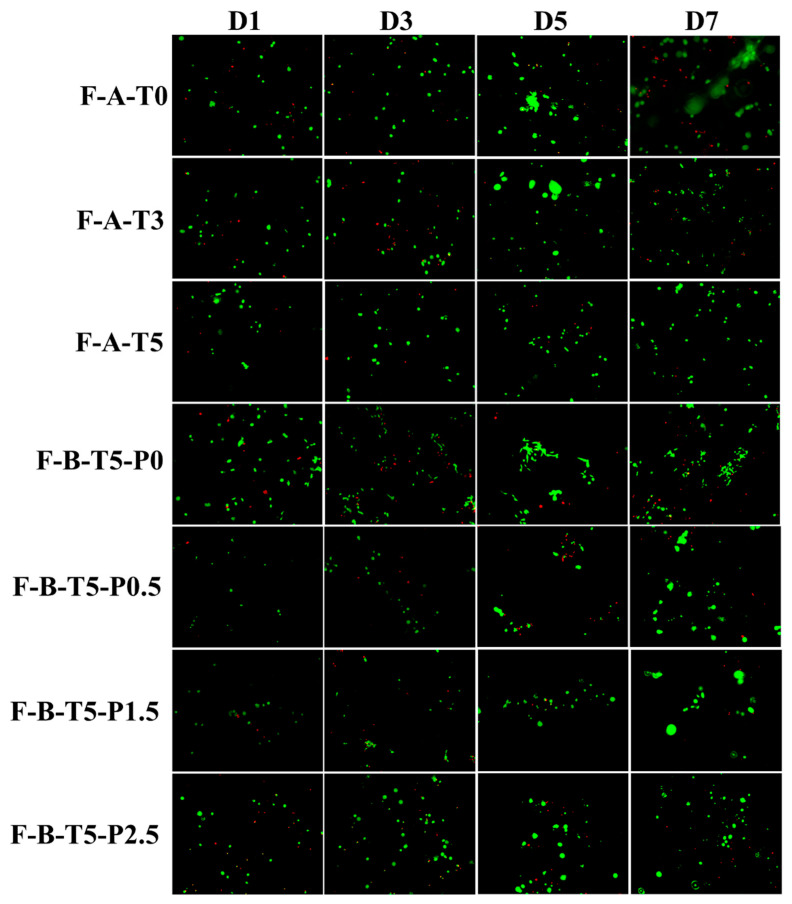
Fluorescence inverted micrograph of MC3T3-E1 cells cultured on F-A-T0/T3/T5 and F-B-P0/P0.5/P1.5/P2.5 (live cells are stained green and dead cells are stained red).

Calcein-AM/PI was used to stain live/dead cells to observe cell survival, as shown in Figure 7. With the extension of the culture time, the number of live cells in the scaffolds increased significantly, and the cells proliferated more efficiently on all scaffolds, which was consistent with the results of CCK-8. Compared with other scaffolds, F-B-T5-P0 showed better ability for cell proliferation and differentiation, but further animal experiments are needed to verify their ability to promote angiogenesis and cell differentiation.

## 4. Conclusions

In this study, we successfully prepared two types of organic–inorganic composite scaffolds (F-A-T0/T3/T5 and F-B-T5-P0/P0.5/P1.5/P2.5). Compared with F-A-T0/T5 and P-A-T3, F-A-T3 exhibited a more uniform distribution of pore sizes (50–150 μm), a rougher surface morphology, and superior mechanical properties. In F-B-T5-P0/P0.5/P1.5/P2.5 scaffolds, the surface roughness, pore size distribution, porosity, and swelling rate decreased with the increase in PVA concentration, while the degradation rate increased. Due to the covalent bonding between PVA and NSiO_2_ and the high viscosity of PVA itself, a dense network structure was formed among CSNF, NSiO_2,_ and PVA, which significantly enhanced the mechanical properties of the scaffolds. The compressive strength of F-B-T5-P1.5 is the highest, with a dry Young’s modulus of 8.53 ± 0.43 MPa and a wet Young’s modulus of 237.78 ± 8.86 kPa, which is four times that of F-A-T5 and double that of F-B-T5-P0. Furthermore, both F-A-T3 and F-B-T5-P1.5 exhibited favorable cytocompatibility, which aided in the attachment and proliferation of MC3T3-E1 cells, but further animal experiments were needed to verify their biomechanical strength. Theoretically, the two types of composite scaffolds developed in this study have significant applications in BTE.

## Figures and Tables

**Table 1:** Composite scaffolds composition.

Sample Code	Chitosan, 2.5% (*w*/*w*)	Hydrolysis Type	TEOS Concentration% (*w*/*w*)	PVA Concentration% (*w*/*w*)
	Types of Chitosan			
P-A-T3	poder	NH_4_OH (A)	3	—
F-A-T0	fiber	NH_4_OH (A)	0	—
F-A-T3	fiber	NH_4_OH (A)	3	—
F-A-T5	fiber	NH_4_OH (A)	5	—
F-B-T5-P0	fiber	HCl (B)	5	0
F-B-T5-P0.5	fiber	HCl (B)	5	0.5
F-B-T5-P1.5	fiber	HCl (B)	5	1.5
F-B-T5-P2.5	fiber	HCl (B)	5	2.5

## Data Availability

Data are contained within the article.
